# Preclinical Studies to Evaluate the Gut Stimulatory Activity of *Aloe Musabbar*

**DOI:** 10.1155/2022/4163008

**Published:** 2022-06-26

**Authors:** Vijayamahantesh K. Tandur, Mohammed Naseeruddin Inamdar, Raha Orfali, Syed Mohammed Basheeruddin Asdaq, Syed Imam Rabbani

**Affiliations:** ^1^Department of Pharmacology, Al-Ameen College of Pharmacy, Bangalore, India; ^2^Department of Pharmacognosy, College of Pharmacy, King Saud University, PO Box 2457, Riyadh 11451, Saudi Arabia; ^3^Department of Pharmacy Practice, College of Pharmacy, AlMaarefa University, Dariyah, 13713 Riyadh, Saudi Arabia; ^4^Department of Pharmacology and Toxicology, College of Pharmacy, Qassim University, Buraydah 51452, Saudi Arabia

## Abstract

**Background:**

Constipation is a common functional gastrointestinal disorder. Medicines derived from nature are routinely used to treat it. The present study evaluates the gut stimulatory activity of *Aloe musabbar* (processed powder of *Aloe vera*) using *in vitro* and *in vivo* models for gut stimulatory activity.

**Materials and Methods:**

*In vitro* tests were conducted on isolated rat colon, guinea pig ileum, and rabbit jejunum, while *in vivo* study was performed using mice intestinal transit time. *Aloe musabbar* (*A*. *musabbar*) was tested at doses 0.2–200 mg/mL (*in-vitro* study) and 86.6 mg/kg (*in vivo* study). *In vitro* studies were done in the presence and absence of atropine sulphate (1 ng/ml). The results were statistically analyzed, and *p* < 0.05 was considered to indicate the significance.

**Results:**

*A*. *musabbar* exhibited dose-dependent increase in the smooth muscle contraction of isolated gut tissues. Presence of atropine minimized the contractile responses and shifted the dose-response curves towards the right-hand side. The intestinal transit time in mice was observed to be increased significantly (*p* < 0.01) in *A*. *musabbar*-treated animals, when compared with normal animals.

**Conclusion:**

A mild smooth muscle contraction induced by *A*. *musabbar* suggests that it can stimulate intestinal bowel movement without causing spasms. The diminished responses in the presence of atropine indicated that the gut stimulatory activity could be mediated partially through parasympathetic innervations. More studies are needed to determine the precise mechanism of action including the specific active ingredient responsible for the gut stimulatory activity.

## 1. Introduction

Constipation is one of the universal problems common among people of different races. It is characterized by a variety of bowel symptoms such as difficulty passing stool, hard stool, and a feeling of incomplete evacuation [1]. Epidemiological evidence suggests that constipation is one of the common gastrointestinal problems among the population, and its prevalence in the world was estimated to be 15% [2]. Several etiological factors have been linked to constipation such as altered lifestyle, lack of physical activity, medications, diet, and diseased states. Medications need to be given in few instances when the patients are chronically constipated and suffer from acute abdominal pain [2, 3].

The etiological finding suggest multifactorial causes for constipation and are considered to be the major limiting factor for the clinical efficacy of current conventional treatments. The medications currently used have a single mechanism through which they work to control constipation [1, 2]. At present, several classes of drugs are available to treat constipation and those important among them are emollients, bulk forming, osmotic, and stimulant laxatives. These drugs show their action mainly through single pathway and are also associated with adverse effects [4]. Some of these reactions are reported to be mild, while few such as hydro-electrolyte imbalances and cramps are serious in nature. The findings suggest that there is still scope for better medications, which are safe and effective in the treatment of constipation [5]. Herbal medicine can be tested and tried to complement the shortcomings of the current western medical model. These naturally derived medicines are reported to provide a complete holistic approach, capable of targeting multiple organs and cellular sites [6].

*Aloe vera* belonging to the family Xanthorrhoeaceae is a perennial herb. The plant bears bright yellow tubular flowers and can be found extensively in several regions of the world that have hot and dry climate. The plant has thick leaves that contains mucilaginous gel-like substance and has been extensively used for several biological activities [7]. Some of the important pharmacological properties reported for *A*. *vera* include anti-inflammatory, antioxidant, antidiabetic, antihyperlipidemic, and anticancer effects. The gel-like substance is popular for treating the skin problems such as burns and wounds and is commonly used in the cosmetics for its skin-nourishing properties [8].

The extract of the plant is reported to contain several active ingredients such as vitamins (vitamins A, C, E, and B12), enzymes (amylase, catalase, and peroxidase), minerals (zinc, copper, selenium, and calcium), and phytoconstituents such as aloin, amodin, lupeol, campesterol, and saponins [8, 9]. In an earlier study, the laxative effect of *A*. *vera* leaf extract was tested against loperamide-induced constipation in rats. The tested dose of extract was reported to improve the intestinal motility, increased faecal volume, and normalized body weight in the constipated rats [10]. In another study, the ability of *A*. *vera* extract to increase the intestinal motility in the experimental rats has been linked to the presence of anthraquinone [11].

*Aloe musabbar* (*A*. *musabbar*) is the solid powder obtained after processing the juice of *A*. *vera*. This form of the leaf extract is used in the Ayurveda and Unani system of medicines and is a common ingredient in cosmetic products [12]. To further explore the gut stimulatory activity of *A. musabbar*, the present study was planned. In this study, the effects of *A. musabbar* was evaluated on isolated gut preparation (*in vitro*) of different species such as rat colon, guinea pig ileum, rabbit jejunum and on mice to determine the *in vivo* intestinal transit time.

## 2. Materials and Methods

### 2.1. Animals

Laboratory bred animals were used in the present study. The animals were housed in the standard animal facilities and were provided with food and water *ad labitum*. 48 hours of acclimatization was done in the lab before animals were prepared for the experiment. The Institutional Animal Ethics Committee approved the experimental protocol (KCP/IAEC-20); animals were maintained under standard conditions in an animal house approved by the Committee for the Purpose of Control and Supervision on Experiments on Animals (CPCSEA).

### 2.2. Preparation of Reagents and Drugs

The chemicals and reagents used in the study were of analytical grade. These agents and the pure drugs such as acetylcholine (Lancaster Synthetic) and atropine (Cipla Limited) were procured from regular suppliers of the institute. The powder extract of *A*. *musabbar* was obtained as a gift sample from Natural Remedies (P) Ltd., Bangalore. The extract and drugs were reconstituted freshly in distilled water as the dosage requirements. The physiological salt solutions such as Tyrode solution and modified Ringer solution were prepared as per the procedure described in the literature [13].

### 2.3. Phytochemical Investigation of the Powder Extract of *A*. *musabbar*

The qualitative phytochemical estimation of the powder extract of *A*. *musabbar* was carried out to determine the availability of phytochemicals including phytoseterols, carbohydrates, flavonoids, proteins, tannins, glycosides, steroids, terpenoids, and saponins using standard methods published in the literature [14].

### 2.4. Experimental Protocol

The in *vitro* studies were conducted using the isolated tissues such as rat colon, guinea pig ileum, and rabbit jejunum. The in vivo study was conducted on mice to determine the propulsive gut motility. The detailed procedure of each experiment is described in the following sections.

#### 2.4.1. Isolated Rat Colon Preparation

Wistar rats of either sex weighing 150–200 g were selected and underwent overnight fasting with free access to water. The animal was sacrificed by cervical dislocation under light ether anesthesia. The abdomen was cut open, and the colon was identified. The right flexure, i.e., the sub hepatic region of the colon, where the ascending colon turns to become the transverse colon was cut out and placed in the shallow dish containing modified Ringer's solution. The lumen was gently cleaned, and a 3 cm long tissue was mounted in the organ bath containing modified Ringer's solution (pH 7.4) maintained at 25°C and bubbled with carbogenated air. The preparation was allowed to equilibrate for 45 minutes under 1 g tension. The concentration-dependent responses of *A*. *musabbar* extract as well as acetylcholine was recorded in the absence and presence of atropine sulphate (1 ng/ml) using a 4-channel polyriter (Recorders and Medicare systems (P) Ltd. Ambala) [15]. The dose-response curve (DRC) of acetylcholine (Ach) was tested at concentrations 1 *μ*g/ml to 1 mg/mL. The extract of *A*. *musabbar* was tested at concentrations 0.2 mg to 200 mg/mL.

#### 2.4.2. Isolated Guinea Pig Ileum Preparation

Guinea pigs of either sex weighing 500–750 g were selected and fasted overnight, with free access to water. The guinea pig was sacrificed under light ether anesthesia. The abdomen was cut open, and the caecum was lifted to trace the ileocaecal junction, about 15 centimeters proximal to the ileocaecal portion, and was removed and immediately placed in the watch glass containing Tyrode solution. The mesentry was trimmed, and with gentle care, the content of the ileum was cleaned by passing the Tyrode solution into the lumen by taking precaution to avoid any damage to the gut muscle. The ileum was cut into small segments of 2–3 cm. One segment of the ileum was tied with the thread to the top and bottom ends without closing the lumen and mounted in the organ bath containing Tyrode solution maintained at 32–35°C and bubbled with carbogen. Each segment was hanged to a force transducer. A tension of 1 g was applied, and the tissue was allowed to equilibrate for 30 minutes before adding the drug to the organ bath. The concentration-dependent responses to acetylcholine and *A*. *musabbar* were recorded using a 4-channel polyriter. A contact time of 30 sec and 3 min time cycle were maintained for recording of responses. Atropine sulphate (1 ng/ml) was added to the tissue bath, and the concentration response curve of Ach and the powder extract of *A*. *musabbar* was obtained in the presence of atropine sulphate [16].

#### 2.4.3. Isolated Rabbit Jejunum Preparation

Rabbits of either sex weighing 1–3 kg were selected and starved for 24 hours with free access to water. The rabbit was sacrificed under light ether anesthesia. The abdomen was cut open, and the caecum was lifted to trace the ileocaecal junction. The jejunum was identified, cut, and immediately placed in the watch glass containing Tyrode solution. The mesentry was trimmed, and with gentle care, the content of the lumen was cleaned by pushing Tyrode solution into the lumen by taking precaution to avoid any damage to the gut muscle. The jejunum was cut into small segments of 2–3 cm long. One segment of the jejunum was taken and tied with the thread to the top and bottom ends without closing the lumen and mounted in the organ bath maintained at 32–35°C and bubbled with carbogen. A tension of 1.5 g was applied, and the tissue was allowed to equilibrate for 30 minutes before adding the drug to the organ bath. The responses due to acetylcholine and the extract of *A*. *musabbar* were recorded using a 4-channel polyriter. A contact time of 30 sec and 3 min time cycle were maintained for the recording of responses. The concentration response curve of acetylcholine and *A*. *musabbar* extract was obtained in the presence of atropine sulphate 2 *μ*g [17].

#### 2.4.4. *In Vivo* Intestinal Transit (IT) Using Mice

Passage of charcoal meal through the gastrointestinal tract in mice was used as the parameter for evaluating intestinal motility [18]. Female Swiss albino mice (20–30 g) were randomly divided into three groups containing 6 mice in each group. The animals were fasted for 24 hours prior to the experiment, with free access to water. Group-I (Control) animals were given 20 ml/kg of normal saline orally; Group-II (Standard) received Dulcolax® (Bisacodyl) at 10 mg/kg. Group-III animals received the extract of *A*. *musabbar* at 86 mg/kg. After 15 min of drug administration (oral route), 0.3 ml of a charcoal meal (distilled water suspension containing 10% gum acacia, 10% vegetable charcoal, and 20% starch) was administered orally to each animal. After 20 minutes, animals were sacrificed under light ether anesthesia and the abdomen was cut opened. The distance traveled by the charcoal plug in comparison to the total length of the small intestine was recorded.

### 2.5. Statistics

Results are expressed as mean ± SEM. Statistical significance was assessed using one-way analysis of variance (ANOVA) followed by Tukey multiple comparison tests. *p* < 0.05 was considered significant.

## 3. Results

### 3.1. Phytochemical Analysis

The qualitative preliminary phytochemical studies of dried extract of *A*. *musabbar* showed the presence of carbohydrates, alkaloids, saponins, flavonoids, steroids, terpenoids, and glycosides.

### 3.2. Isolated Rat Colon Preparation

**Figure 1:**
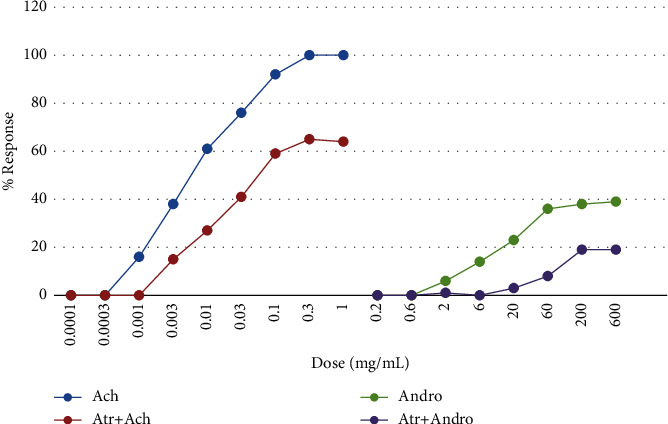
Comparative DRC of Ach and *Aloe musabbar* on isolated rat colon preparation in presence and absence of atropine sulphate. *Note.* Values are represented as mean ± SEM.

**Figure 2:**
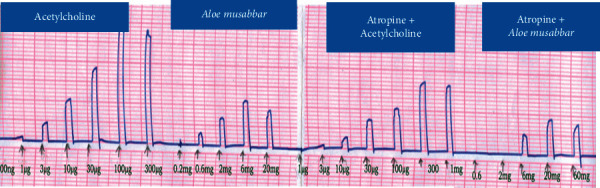
Comparative DRC of Ach and *Aloe musabbar* on isolated rat colon preparation in presence and absence of atropine sulphate.

The observations recorded from the isolated tissue indicated that both Ach and *A*. *musabbar* produced dose-dependent increase in the contractile responses. Saturation in the contractile response was observed for both Ach (1 mg/mL onwards) and *A*. *musabbar* (60 mg/mL). The responses of Ach and *A*. *musabbar* were repeated in the presence of atropine sulphate (1 ng/ml), and the record suggested that the curve moved towards the right-hand side. Besides, both the DRCs were found to be diminished and also showed dose-dependent contraction at lower doses and ceiling effect at higher concentrations (Figures 1 and 2).

### 3.3. Isolated Guinea Pig Ileum Preparation

**Figure 3:**
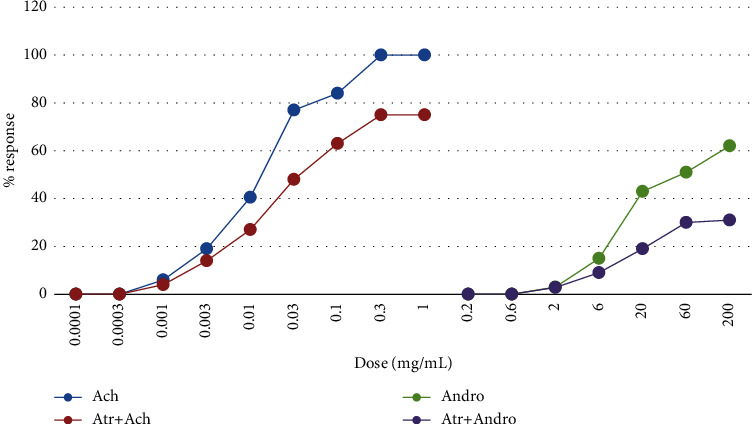
Comparative DRC of Ach and *Aloe musabbar* on isolated guinea pig ileum preparation in presence of atropine sulphate. *Note*. Values are represented as mean ± SEM.

**Figure 4:**
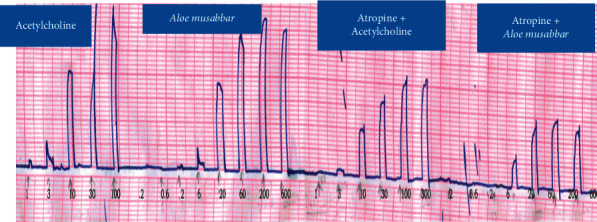
Comparative DRC of Ach and *Aloe musabbar* on isolated guinea pig ileum in presence and absence of atropine sulphate.

Figure 3 represents the responses recorded for Ach and *A*. *musabbar* in the isolated guinea pig ileum. Ach responses were recorded at the concentration 1 ng to 300 *μ*g/ml. Then, responses were tested in the presence and absence of atropine sulphate (1 ng/ml). Dose-dependent increase in the contractile response was recorded till 0.1 mg/mL, and then, saturation in the effect was observed. However, when atropinized tissue was tested, Ach produced dose-dependent increase in contraction at all the tested doses but shifted the DRC towards the right-hand side. The *A*. *musabbar* responses were recorded at the concentration ranging from 0.2 mg to 200 mg/mL. The observations suggested a dose-dependent effect at lower doses, and from 60 mg/mL, a ceiling effect in the contractile response was observed. In the atropinzed tissue (1 ng/ml), the responses of *A*. *musabbar* was found to be diminished and shifted to the right-hand side (Figure 4).

### 3.4. Isolated Rabbit Jejunum Preparation

**Figure 5:**
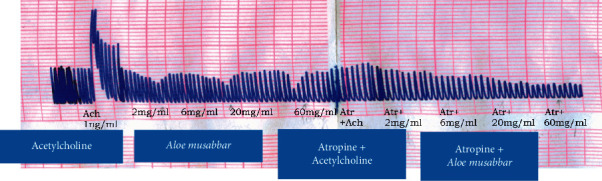
Effect of Ach and *Aloe musabbar* on isolated rabbit jejunum preparation.

The responses recorded with the isolated rabbit jejunum were tested in the presence and absence of atropine as represented in Figure 5. The spontaneous smooth muscle contraction was first recorded with Ach at concentration ranging between 1 and 3 ng/ml. The observations suggested a dose-dependent increase in the tonicity of contraction. In the presence of atropine sulphate, responses of Ach were found to be abolished. *A*. *musabbar* was tested at concentration 2 mg to 60 mg/mL. The administration of the extract increased the pendular movements without increasing the tonicity from the intestinal smooth muscles. Besides, when these concentrations were tested in the presence of atropine sulphate, complete abolition of responses was observed.

### 3.5. Intestinal Transit (IT) in Mice

**Figure 6:**
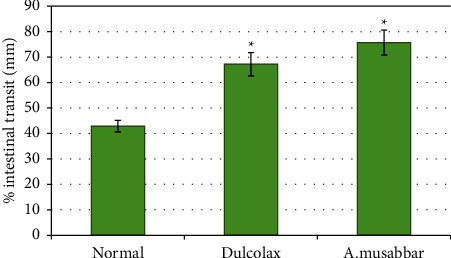
Effect of *Aloe musabbar* extract on the intestinal transit (IT) in mice. *Note*. Values are represented as mean ± SEM. Statistics: one-way ANOVA; ^*∗*^*p* < 0.01 compared to normal.

Figure 6 represents the intestinal transit of charcoal meal recorded for Dulcolax® and *A*. *musabbar* in mice. The average intestinal transit in the control group of animals was found to be 42.9 mm. Dulcolax® (10 mg/mL) used as a standard herbal laxative increased the intestinal transit to 67.19 mm and was found to increase (*p* < 0.01) the distance significantly, when compared with normal group. Furthermore, when *A*. *musabbar* was tested at the dose 86.6 mg/mL, the administration was found to have increased the intestinal transit to 75.73 mm. The data when compared with normal group showed significant (*p* < 0.01) enhancement in the intestinal transit.

## 4. Discussion

In the present study, *A*. *musabbar* was tested for gut stimulatory activity using *in vitro* and *in vivo* experimental animal models (Figures 1, 3, and 6). *A*. *musabbar* is a solid powder obtained after processing the juice of *A*. *vera*. This form of *A. vera* is used in the formulation of medicinal practices under Ayurveda and Unani systems [11]. The powder form of *A*. *vera* is quite famous and commonly used in the preparation of cosmetic products. The dried powder form of *A*. *vera* (i.e., *A*. *musabbar*) is preferred over drug gel due to presence of essential phytoconstituents, and it is devoid of major impurities [12].

*A*. *vera* is known to possess several biological activities including the laxative effect [8, 9]. Isolated gut preparations are used extensively to determine the stimulatory/inhibitory properties on the intestinal smooth muscles [19]. The experimental set-up offers the advantages of maintaining a tight control over chemical and physical environment with the option of repeating the experiments without incurring extra cost and using animals [18]. However, because of the major disadvantage of not replicating the conditions of cells in an organism, an in vivo intestinal transit in mice was studied to evaluate the gut stimulatory property in the present study [20]. The observations suggested that the powder extract of *A*. *musabbar* produced a dose-dependent stimulatory action on the isolated rat colon and guinea pig ileum (Figures 1 and 3). Acetylcholine used as a standard spamogenic agent showed potent gut stimulatory activity recorded in terms of higher contraction of smooth muscles at lower concentrations (Figures 2 and 4). Studies conducted in the past have shown similar observations. In one such study, it was reported that the extract of *Asparagus cochinchinesis* produced gut-stimulatory activity in the transverse colon of Sprague-Dawley rats [21].

The studies conducted on the isolated rabbit jejunum suggested that both Ach and *A*. *musabbar* showed increased pendular movements in the smooth muscles. However, the action of Ach was found to be associated with increased tonicity of the smooth muscle contraction (Figure 5). Pendular movements are reported to be the normal rhythmic contraction and relaxation seen in the jejunum during normal digestion process. However, increased tonicity of smooth muscles observed with Ach could be the cause for smooth muscle spasms [21]. A study conducted on the methanolic extract of *Viola betonicifolia* indicated that ingestion of the whole plant increased the pendular movements in the jejunum. These actions were reported to relieve constipation, and the mechanism for this has been partially linked to the alkaloidal and saponin content of the extract, mediated through the parasympathetic innervations [22]. As a result, *A*. *musabbar*'s alkaloids and saponins may have contributed to this parasympathomimetic effect.

The findings from isolated gut preparations indicated that in the presence of atropine sulphate, the responses of both Ach and *A*. *musabbar* diminished and the dose-response curves shifted to the right-hand side (Figures 1 and 3). Furthermore, the magnitude of smooth muscle contractions in the rat colon and guinea pig ileum (Figures 2 and 4) and the pendular movements in the rabbit jejunum were also reduced in the presence of atropine sulphate (Figure 5). These observations suggested that the response of *A*. *musabbar* on the isolated tissues could be partly mediated through parasympathetic transmission. Similar observations were reported when the *Viola betonicofolin* extract was tested in isolated rabbit and guinea pig gut preparation to measure the isotonic contraction [22].

The available evidence suggests that parasympathetic innervations play an important role in the gut motility [23]. Studies have also indicated that Ach stored in the nonneuronal cells of the gut plays an important role in stimulating the smooth muscle contraction [24]. Furthermore, the muscarinic receptor innervations can also increase the secretion of mucus from goblet cells. The mucus in the gut lubricates the content and assists in intestinal expulsion of faecal matters [25]. The comparison of the responses of Ach and *A*. *musabbar* suggested that both the compounds stimulated the gut smooth muscles (Figures 2, 4, and 5) but the contraction induced by Ach might be spasmogenic in nature [21]. On the other hand, the smooth muscle contraction induced by *A*. *musabbar* was found to be mild. Besides, the increased pendular movements of the jejunum observed with *A*. *musabbar* could benefit in churning and mixing of luminal contents essential for optimum digestion process [22].

The intestinal transit distance estimation in mice indicated that *A*. *musabbar* treatment increased (*p* < 0.01) the intestinal transit distance significantly, when compared to the normal animals. Similarly, the standard herbal drug (Dulcolax®) also produced significant (*p* < 0.01) increase in the intestinal transit distance in comparison with control animals (Figure 6). The intestinal transit signifies the distance a charcoal meal travels in the intestine after oral administration [25]. In an earlier study, the increased intestinal transit time observed with *Asparagus cochinchinesis* in the Sprague-Dawley rats has been linked to the saponin contents of the herbal medicine [22]. Therefore, it is possible that the saponins of *A*. *musabbar* played a role in increasing intestinal transit distance. Constipation is a common gastrointestinal problem and chronic sufferers of it are reported to develop haemorrhoids, anal fissure, faecal impactions, and rectal prolapse [2, 3]. *A*. *musabbar* being an herbal medicine was reported to be used in the treatment of several diseases. The important phytochemical constituents of *A*. *vera* leaf include alkaloids, flavonoids, saponin, phenol, glycosides, and tannins [8, 9]. Presence of alkaloid and saponin components in the earlier studies have been linked to the parasympathetic properties [9, 21, 26] and could most likely be responsible for increasing the smooth muscle contractions observed in the present study. Being a known component of several systems of medicine, *A*. *musabbar* could become a potential agent for treating the gut-motility associated disorders, provided its precise mechanism is established and active constituents are identified.

## 5. Conclusion

Analysis of the present study data indicated the gut stimulatory activity of *A*. *musabbar* in *in vitro* and *in vivo* experimental models. The actions of *A*. *musabbar* appear to be mediated partly through parasympathetic activation. Being a known herbal drug in several systems of medicines, *A*. *musabbar* could be a potential agent for treating the gut-motility-associated defects. However, more research is suggested to establish the precise mechanism of action and active phytoconstituents responsible for gut stimulatory effect.

## Data Availability

The data are available on request to the corresponding author.
